# A prognostic model to personalize monitoring regimes for patients with incidental asymptomatic meningiomas

**DOI:** 10.1093/neuonc/noz160

**Published:** 2019-10-11

**Authors:** Abdurrahman I Islim, Ruwanthi Kolamunnage-Dona, Midhun Mohan, Richard D C Moon, Anna Crofton, Brian J Haylock, Nitika Rathi, Andrew R Brodbelt, Samantha J Mills, Michael D Jenkinson

**Affiliations:** 1 Institute of Translational Medicine, University of Liverpool, Liverpool, UK; 2 School of Medicine, University of Liverpool, Liverpool, UK; 3 Department of Clinical Oncology, The Clatterbridge Cancer Centre NHS Foundation Trust, Wirral, UK; 4 Department of Neurosurgery, The Walton Centre NHS Foundation Trust, Liverpool, UK; 5 Department of Neuroradiology, The Walton Centre NHS Foundation Trust, Liverpool, UK; 6 Department of Neuropathology, The Walton Centre NHS Foundation Trust, Liverpool, UK

**Keywords:** asymptomatic, incidental, meningioma, prognosis, risk score

## Abstract

**Background:**

Asymptomatic meningioma is a common incidental finding with no consensus on the optimal management strategy. We aimed to develop a prognostic model to guide personalized monitoring of incidental meningioma patients.

**Methods:**

A prognostic model of disease progression was developed in a retrospective cohort (2007–2015), defined as: symptom development, meningioma-specific mortality, meningioma growth or loss of window of curability. Secondary endpoints included non-meningioma-specific mortality and intervention.

**Results:**

Included were 441 patients (459 meningiomas). Over a median of 55 months (interquartile range, 37–80), 44 patients had meningioma progression and 57 died (non-meningioma-specific). Forty-four had intervention (at presentation, *n =* 6; progression, *n =* 20; nonprogression, *n =* 18). Model parameters were based on statistical and clinical considerations and included: increasing meningioma volume (hazard ratio [HR] 2.17; 95% CI: 1.53–3.09), meningioma hyperintensity (HR 10.6; 95% CI: 5.39–21.0), peritumoral signal change (HR 1.58; 95% CI: 0.65–3.85), and proximity to critical neurovascular structures (HR 1.38; 95% CI: 0.74–2.56). Patients were stratified based on these imaging parameters into low-, medium- and high-risk groups and 5-year disease progression rates were 3%, 28%, and 75%, respectively. After 5 years of follow-up, the risk of disease progression plateaued in all groups. Patients with an age-adjusted Charlson comorbidity index ≥6 (eg, an 80-year-old with chronic kidney disease) were 15 times more likely to die of other causes than to receive intervention at 5 years following diagnosis, regardless of risk group.

**Conclusions:**

The model shows that there is little benefit to rigorous monitoring in low-risk and older patients with comorbidities. Risk-stratified follow-up has the potential to reduce patient anxiety and associated health care costs.

**Key Points**

1. Most incidental meningiomas do not progress during follow-up.
2. Risk of incidental meningioma progression plateaus after 5 years of follow-up.
3. Baseline imaging and clinical factors can be used to guide personalized monitoring.

**Importance of the Study**

Incidental meningioma is common, with no consensus on the optimal management strategy. International guidelines recommend monitoring with MRI for managing these tumors; however, details regarding the optimal duration and intervals for follow-up are lacking. This often prompts clinicians to commence long-term follow-up, which is of uncertain patient benefit and has economic implications. Using data from 441 patients with incidental meningiomas, we developed a prognostic model which can be used to predict an individualized disease progression risk and tailor monitoring. Our study showed that most incidental meningiomas remain stable during follow-up and that growth plateaus after 5 years. Tumor hyperintensity, increasing meningioma volume, proximity to critical neurovascular structures, and peritumoral signal change all increase the risk of disease progression within the first 5 years following diagnosis. To aid clinical decision making, these imaging factors, alongside patient age, comorbidity, and performance status, were used to build the IMPACT calculator, freely available to clinicians (www.impact-meningioma.com).

Wider access and increased use of brain imaging have led to a marked rise in the number of incidental findings in clinical and research settings, including meningiomas.^1^ Incidental meningiomas cause patient anxiety and uncertainty around the need for future treatment and often prompt clinicians to commence long-term follow-up. International consensus guidelines suggest active monitoring with MRI as first line for managing these tumors^2^; however, data to support the optimal duration and intervals for follow-up are lacking.^3^ Several studies have identified prognostic imaging factors that are associated with the risk of meningioma growth and development of clinical symptoms^4,5^; however, the timing of such progression is poorly defined. Moreover, clinical factors such as patient comorbidity and performance status remain unexplored in relation to prognosis but are equally important for clinical decision making. Patients with an incidental meningioma want to know whether their tumor will grow and become symptomatic such that it will require (safe) treatment within their (healthy) lifetime. The aim of this study was to combine routinely available imaging and clinical factors to develop a prognostic model for the risk of incidental meningioma progression during active monitoring.

## Materials and Methods

### Study Design

We performed a retrospective cohort analysis of adults (age ≥16 y) with a newly identified incidental asymptomatic meningioma between January 2007 and December 2015, with follow-up through to March 2018. Patients with radiation-induced and neurofibromatosis type 2–associated meningiomas and with incomplete medical records were excluded. The study setting was the Walton Centre NHS Foundation Trust, the only specialist stand-alone neuroscience hospital in the UK. It serves a catchment area of 3.5 million people and has service partnerships with 18 other hospitals. The institutional review boards at the authors’ institutions approved this study.

### Study Endpoints

#### The primary composite endpoint was

Symptom development, meningioma-specific mortality, development or increase of peritumoral signal intensity (vasogenic edema), venous sinus invasion, or meningioma volume exceeding 10 cm^3^. The first 2 criteria denote clinical progression, while the latter 3 are related to loss of a window of curability. Venous sinus invasion and peritumoral edema can prevent complete surgical resection.^6,7^ Peritumoral edema and a meningioma volume >10 cm^3^ are relative contraindications to stereotactic radiosurgery.^8,9^

#### The secondary endpoints were

The occurrence of an intervention and mortality unrelated to the meningioma.

### Baseline Predictive Variables

Patient age, sex, World Health Organization performance status (PS),^10^ and age-adjusted Charlson comorbidity index (ACCI)^11,12^ were derived from the medical records. Imaging variables assessed were: (i) number of meningiomas, (ii) calcification on noncontrast computed tomography (CT) (diffuse/partial/absent), (iii) tumor signal intensity compared with the contralateral gray matter on T2-weighted or fluid attenuated inversion recovery (FLAIR) MRI (hypo/iso/hyper), (iv) peritumoral signal intensity in relation to tumor volume using the signal change present on T2/FLAIR MRI (0–5%/6–33%/34–66%/67–100%^13^), and (v) meningioma volume using the ABC/2 formula on contrast-enhanced T1-weighted MRI/CT: (A) maximum meningioma diameter on axial plane, (B) diameter perpendicular to (A), and (C) maximum height on coronal/sagittal plane. Meningioma location was classed into non–skull base and skull base and further subcategorized according to the International Consortium on Meningioma (ICOM) classification system.^3^ Meningiomas in proximity to major dural venous sinuses (superior sagittal/transverse/sigmoid/cavernous/torcula) were categorized as separate (≤10 mm), in direct contact with its wall or invading. Contact with critical neurovascular structures (eg, optic apparatus) was noted. Meningiomas that fulfilled one of the two previous categories were said to be in proximity to critical neurovascular structures. Inter- and intra-observer reliability of imaging parameters were assessed on a random sample of 24 patients (sample size determined using the Bland equation^14^) by 2 observers (A.I.I. and M.M.) using weighted Cohen’s kappa or the intraclass correlation coefficient as appropriate.

### Statistical Analysis

Two series of analyses were undertaken—firstly, to determine an appropriate definition of meningioma growth, and secondly to inform the prognostic model. Where appropriate, differences across groups were explored with the χ2 test for categorical variables and a one-way analysis of variance or Kruskal–Wallis test for continuous variables. Normally distributed variables were expressed as mean (standard deviation [SD]), whereas skewed variables were expressed as median (interquartile range [IQR]). Correlation between baseline variables was evaluated using the Pearson correlation coefficient. Differences were considered statistically significant at *P* < 0.05. Analyses were performed using R v3.5.0 and SPSS v24.0.

#### Meningioma growth definition

There is no agreed standard definition of meningioma growth.^15^ For standardization across untreated incidental meningiomas, we used existing measures: extent of growth and annual growth rate.^3^ To determine which is most appropriate, we conducted a series of analyses to examine the temporal relationship between disease progression and meningioma volume.

The association between baseline variables and the initial composite disease progression endpoint was assessed using Kaplan–Meier (KM) analysis. Statistical significance was examined using the log-rank test. Patients who did not experience disease progression and remained under observation were censored at the last recorded follow-up. Patients discharged from outpatient care, died during follow-up, or were lost to follow-up were censored at the last date of follow-up, where there was no evidence of disease progression.

To determine how longitudinally changing meningioma volume is associated with the hazard for disease progression, a joint longitudinal and time-to-event model was fitted. The longitudinal submodel comprised a linear mixed-effect regression model for meningioma volume (natural logarithm) and included both the random intercept and slope. The survival submodel comprised a time-varying covariate semi-parametric Cox proportional hazards model, which included patient level meningioma volume predicted from the longitudinal submodel. The final joint model included baseline variables with *P* ≤ 0.10. Standard errors and *P*-values of the estimated model parameters were obtained using 200 bootstrap samples.

Extent of growth or annual growth rate definitions, based on the statistical effect of time, were examined in relation to our initial criteria of disease progression. A classification and regression tree (CART) analysis was used to assess the degree of success by which these definitions can set our cohort apart stratified by disease progression.

#### Prognostic model

KM analysis, using initial composite endpoint and adopted meningioma growth definition, was performed as described above. A Cox regression model was subsequently developed. Backward and forward stepwise selection procedures were utilized to determine the model of best fit, with covariate inclusion at *P* ≤ 0.05 and exclusion at *P* ≥ 0.10. Skewed continuous variables were transformed into their natural logarithms before being input into the model. Certain covariates were included despite being statistically nonsignificant due to their clinical importance.

A prognostic index was developed based on the results of the Cox model. This was calculated for each patient as the sum of the covariate values included in the final model, weighted by the natural logarithmic transformation of the hazard ratios.

Risk group stratification was carried out by visual assessment of a prognostic index histogram. The prognostic index for each patient was plotted along the *y*-axis, while the frequencies of observed disease progression and nonprogression were plotted on the *x*-axis. Wherever a noticeable increase in the proportion of disease progression occurred in relation to the frequency of nonprogression, a cutoff line was drawn. This was carried out twice to best separate the study cohort into 3 distinct risk groups: low, medium, and high risk. The probabilities of progression-free survival by 0.5, 1, 2, 3, 4, 5, 6, 7, 8, 9, and 10 years were then calculated for each of these groups with KM analysis used to assess differences across them.

Model assumptions were examined using Schoenfeld residuals, and bootstrapping was performed to assess its internal validity (with 200 samples). Calibration was assessed using plots of observed versus predicted disease progression at 5 and 10 years following diagnosis in sextiles of predicted risk. Discrimination was assessed using Harrell’s concordance statistic and Chambless and Diao’s time-dependent area under the receiver operating characteristic curve.^16,17^

The effects of patient age, comorbidity, and PS on the risk of disease progression and intervention were assessed in a competing risk analysis. Patients with normal (PS 0) or limited activity who were ambulatory and able to carry out light work (PS 1) at the time of diagnosis were grouped and compared against ambulatory patients capable of all self-care but unable to carry out any work activities (PS 2), those in a chair/bed for ≥50% of the day but not bedridden (PS 3), and bedridden patients (PS 4). Patients were also stratified by ACCI into: 0–2 (young patients with few or no comorbidities), 3–5 (older patients with few comorbidities or younger patients with several comorbidities), and ≥6 (older patients with comorbidities).^18^

Two competing risk analyses were performed. One assessed the cumulative incidence rate (CIR) of intervention following diagnosis stratified by ACCI and PS groups. The other evaluated the CIR of disease progression. The competing event for the former was non-meningioma-specific mortality, which was observed either during follow-up or after discharge from outpatient care. Patients who remained under follow-up were censored at the last outpatient clinic appointment. Patients discharged alive from outpatient care were censored at the last time they were seen by a health care physician. For the disease progression analysis, competing events were: discharge from outpatient care, loss to follow-up, death during follow-up, or an intervention before disease progression occurred, with the first three grouped together. Censoring was done for patients who remained under follow-up at the last clinic appointment. The Fine and Gray test was carried out to test equality across groups.

### Additional Analyses

Due to the lack of a standardized surveillance protocol at our center, the growth rate for each meningioma was determined using a linear mixed model, which does not require regularly spaced time points, assuming a different intercept and slope for each meningioma. Absolute growth rate (AGR) was defined as the increase in volume per year in cubic centimeters, whereas relative growth rate (RGR) was defined as the percentage increase in volume per year.

## Results

### Study Population and Baseline Characteristics

A total of 441 patients were included (Supplementary Fig. 1); 18.5% of all meningioma patients identified and 9.10% of incidental neurological findings. The number of patients identified per year increased in a linear fashion (Supplementary Fig. 2). Meningiomas were solitary in 426 patients and multiple in 15, resulting in an overall meningioma population of 459. Baseline characteristics are summarized in Supplementary Table 1.

### Treatment Arms and Outcomes

At initial presentation, 6 patients underwent surgical resection, 50 were discharged, and the remaining 385 patients (403 meningiomas) commenced active monitoring (median 36.0 mo; IQR 18.0–57.0). Differences in baseline characteristics across the treatment groups are shown in Supplementary Table 1. The total number of scans performed following diagnosis in the active monitoring group was 1303 (3.4/patient); 1166 had MRI, while the remainder had CT. Most patients (*n =* 360) were consistently monitored using the same imaging modality: MRI in 317 patients and CT in 43. The remaining 25 patients were followed up alternately with CT and MRI. Overall outcomes by the end of the study period were: discharged (*n =* 219), under continued observation (*n =* 205), lost to follow-up (*n =* 12), and deceased during follow-up (unrelated to the meningiomas) (*n =* 5). Records for patients discharged or lost to follow-up were examined (median 34.0 mo; IQR 20.0–56.0) and 52 patients died after a median of 18.5 months (IQR 11.3–37.0) following termination of follow-up. The median overall follow-up duration was 55.0 months (IQR 37.0–80.0).

### Meningioma Growth Endpoint

The joint model showed that time is strongly associated with the initial composite endpoint (*P* < 0.001) (Supplementary Tables 2 and 3) and since meningioma growth is likely to precede these endpoints, and certain factors such as surgical intervention might have prevented their occurrence, it is reasonable that survival analyses incorporate tumor volume change over time (annual rate) as an additional endpoint. The CART analysis for the growth endpoint AGR ≥ 2 cm^3^/year OR AGR ≥ 1 cm^3^/year+RGR ≥ 30%/year^19^ demonstrated a superior misclassification rate and improvement score to other time-dependent growth definitions (see Supplementary Figures 3 and 4). Therefore, disease progression in our study was defined using the initial composite endpoint in addition to the aforementioned growth endpoint.

### Disease Progression and Intervention

**Fig. 1:**
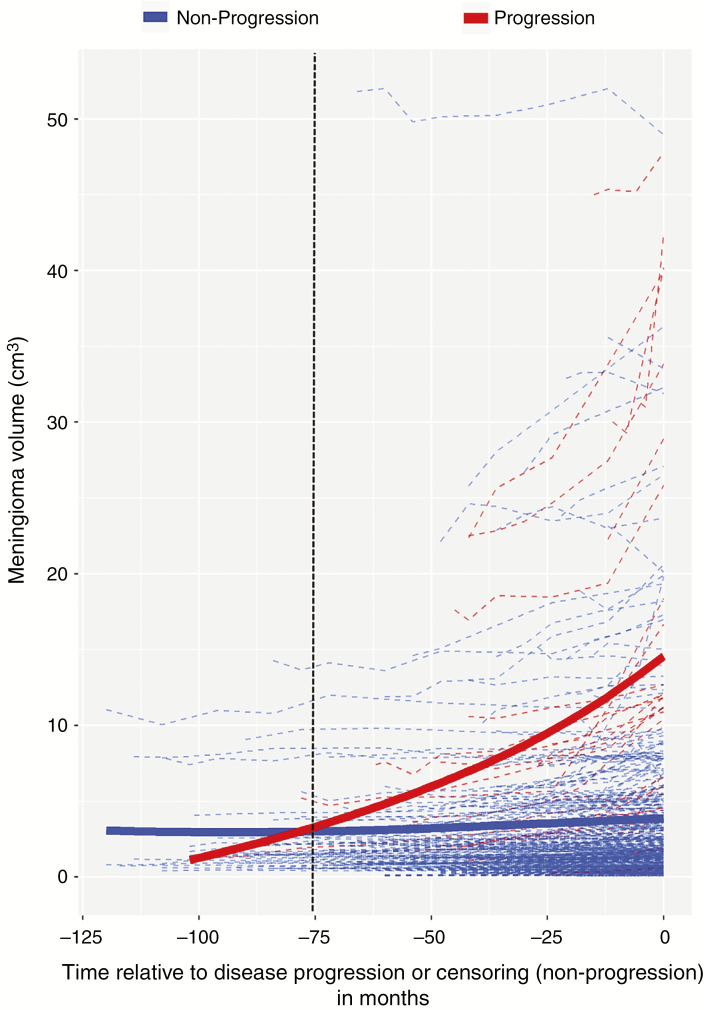
Profile plot for meningioma volume against reverse time stratified by disease progression status. Bold curves are LOESS (locally fitted estimated scatterplot smoothing) curves. While incidental meningiomas that did not progress remained static in size during follow-up, meningiomas that did progress exponentially grew prior to reaching a disease progression endpoint. The time course over which disease progression occurred is denoted by the dotted intersection line. It shows that if 2 equally sized meningiomas were picked up at the same point in time, the meningioma with growth potential will reach its disease progression endpoint by the 75th month (~6th y) following diagnosis.

During follow-up, 44 (11.4%) patients had meningioma progression. Endpoints included: meningioma growth (*n =* 29), new symptom development (*n =* 12), increase in peritumoral signal change (*n =* 10), meningioma volume exceeding 10 cm^3^ (9/369 with an initial volume <10 cm^3^), and venous sinus invasion (5/137 adjacent to but not invading a sinus). Symptoms were seizure (*n =* 6), motor deficit (*n =* 3), visual deficit (*n =* 2), and ataxia (*n =* 1). Twenty-eight experienced one disease progression endpoint, whereas 16 had multiple (12 patients, *n =* 2; 3 patients, *n =* 3; 1 patient, *n =* 4). Median time to disease progression was 33.0 months (IQR 15.0–46.5). The 5- and 10-year progression-free survival rates were 83.0% (95% CI: 77.1–88.9) and 70.0% (95% CI: 56.3–83.7), respectively. The mean longitudinal profiles for meningioma volume against time relative to disease progression are shown in Fig. 1; if 2 equally sized meningiomas were detected at the same point in time, the meningioma with growth potential will have reached its disease progression endpoint by the 75th month following diagnosis.

Rates of intervention and its prerequisite recommendation were significantly lower in the nonprogression group (Table 1; *P* < 0.001). In the disease progression group, an intervention was recommended in 37 patients but carried out in only 20. Median time to intervention in both cohorts was 24.0 months (IQR 11.8–42.0).

**Table 1.** Differences in growth dynamics and intervention outcomes between the progression and nonprogression groups

Characteristic	Disease Progression	Nonprogression	*P*
	(*N* = 44)	(*N* = 359)	
Median AGR/year in cm^3^ (IQR)	1.36 (0.72–2.58)	0.05 (0.01–0.17)	<0.001^*a*^
Median RGR/year in % (IQR)	26.7 (14.5–38.8)	4.13 (0.81–8.39)	<0.001^*a*^
Intervention recommended, *N* (%)	37 (84.1)	16 (4.46)	<0.001^*b*^
Intervention, *N* (%)	20 (45.5)	18 (5.01)	<0.001^*b*^
Intervention as per patient request, *N* (%)	0 (0.00)	6 (1.67)^*c*^	0.789^*b*^

^*a*^Kruskal–Wallis test.

^*b*^χ2 test.

^*c*^Requested surgery after a median follow-up period of 4.5 months (IQR 3.0–15.0).

When treatment was offered for imaging reasons alone (disease progression group, *n =* 11; nonprogression group, *n =* 4), patients tended to decline, since they were clinically stable. Disease progression in 6 patients additionally involved new symptom development, which patients either elected to control with anti-epileptics (seizure, *n =* 5) or were happy to live with due to minimal impact on quality of life (visual field deficit, *n =* 1). Of the 12 patients who progressed and had further imaging surveillance available, 11 continued to show evidence of meningioma growth (median follow-up period after initial disease progression 21.0 mo; IQR 13.5–24.0). Three patients with epilepsy had controlled seizures at their last follow-up, despite continued meningioma growth in 2 patients (mean follow-up period after initial disease progression 16.0 mo [SD = 2.8]).

### Prognostic Model

KM analyses (Supplementary Table 4) revealed male sex (*P* = 0.005), increasing tumor volume (*P* < 0.001), absence of calcification (*P* < 0.001), peritumoral signal change (*P* < 0.001), and T2/FLAIR hyperintense meningioma (*P* < 0.001) to be significantly associated with disease progression. Following backward stepwise regression analysis (Table 2; model 1), 2 prognostic factors were identified: T2/FLAIR hyperintense meningioma, and meningioma volume (natural logarithm). Absence of calcification was not included in the model as hypointensity on T2/FLAIR acts as a surrogate for calcification on CT (bivariate correlation, *P* < 0.001). Forward stepwise regression was subsequently performed to examine the prognostic importance of variables with a significance level of *P* > 0.10, together with interaction terms of prognostic factors identified in the first model and variables excluded from the first analysis. No additional factors were identified. Two imaging parameters were, however, deemed clinically important and were included in the model, namely proximity to critical neurovascular structures and peritumoral signal change (Table 2; model 2).

**Table 2.** Hazard ratios (95% CI) of statistically and clinically important factors in multivariate analysis

	Model 1^*a*^		Model 2	
Factor	HR (95% CI)	*P*	HR (95% CI)	*P*
Meningioma volume (natural logarithm)	2.43 (1.82–3.24)	<0.001	2.17 (1.53–3.09)	<0.001
Meningioma hyperintensity	11.2 (5.72–21.9)	<0.001	10.6 (5.29–21.0)	<0.001
Peritumoral signal change	–	–	1.58 (0.65–3.85)	0.313
Proximity to critical neurovascular structures	–	–	1.38 (0.74–2.56)	0.314

^*a*^Results of the backward stepwise regression, investigating the set of variables with a log-rank *P* ≤ 0.10.

**Fig. 2:**
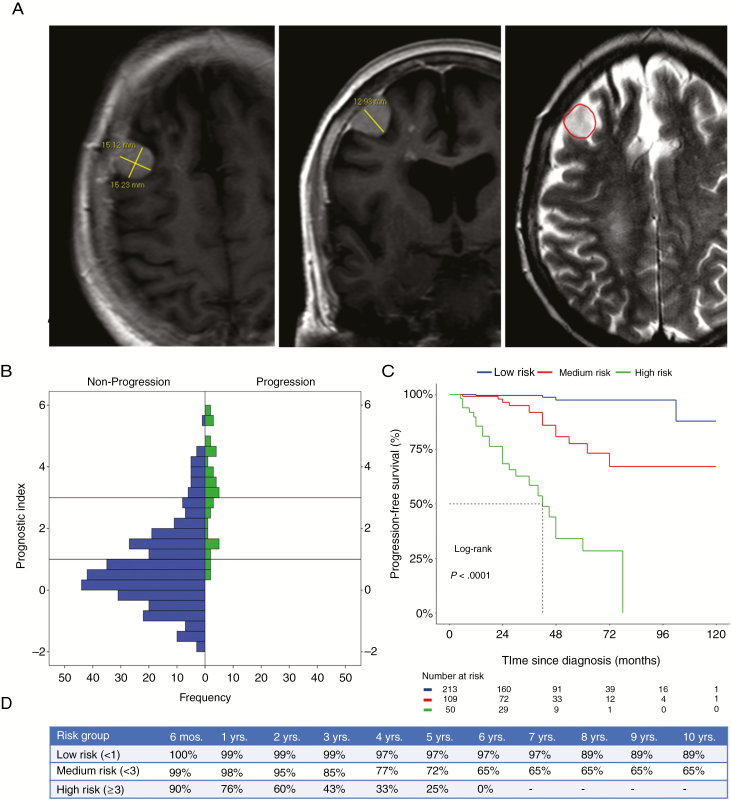
(A) A 1.50 cm^3^ hyperintense convexity meningioma distant from critical neurovascular structures unaccompanied by peritumoral signal change. Using the prognostic index (LN1.50×LN2.17) + (1×LN10.6) + (0×LN1.58) + (0×LN1·38) = 2.8, this meningioma could be classified as medium risk. (B) Histogram of the disease progression and nonprogression cases plotted against the prognostic index demonstrating the 2 cutoff lines. (C) KM plot stratified by risk group. (D) Table with progression-free survival probabilities at different time points following diagnosis stratified by risk group. LN = natural logarithm.

Based on the results of model 2, a prognostic index (Fig. 2A) was generated for each patient and plotted against the observed frequencies of progression and nonprogression in a histogram (Fig. 2B). Risk group stratification was performed by visual assessment and appropriate partitioning by cutoff points, allowing for the creation of 3 distinct risk groups: low risk (<1), medium risk (<3), and high risk (≥3). KM analysis (Fig. 2C) demonstrated a significant difference (*P* < 0.001) in the probabilities of progression-free survival (Fig. 2D) following diagnosis across risk groups.

**Fig. 3:**
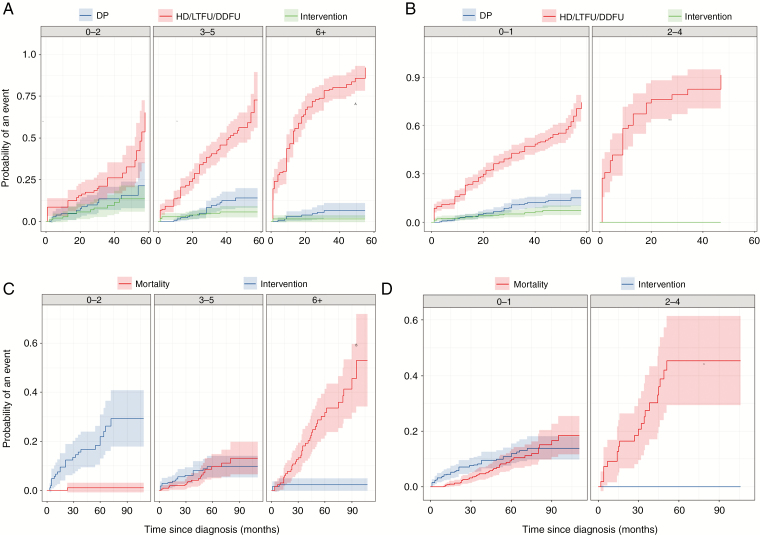
(A–B) Estimated cumulative incidence curves (solid lines) for disease progression and its competing events with 95% CIs (shading) stratified by (A) ACCI and (B) PS. (C–D) Estimated cumulative incidence curves (solid lines) for intervention and mortality with 95% CIs (shading) stratified by (C) ACCI and (D) PS. DP: disease progression; DDFU: deceased during follow-up; HD: hospital discharge; LTFU: lost to follow-up.

CIR plots of disease progression and intervention are shown in Fig. 3 (and Supplementary Tables 5 and 6). Stratified by ACCI, the rates of intervention were statistically different across the 3 groups (*P* < 0.001), although the rates of disease progression were not (*P* = 0.090). Approximately 80% of patients with an ACCI ≥6 were discharged, deceased, or lost to follow-up at 5 years following diagnosis, having not had disease progression. Patients with an ACCI ≥6 were also 15 times more likely to die within 5 years of follow-up than to receive an intervention. Patients with an ACCI of 0–2 were 3 times more likely to have experienced disease progression at 5 years compared with patients with an ACCI ≥6. The rates of intervention and mortality did not differ in patients with an ACCI of 3–5. Differences in incidence rates of disease progression and intervention among the PS groups were statistically significant (*P* < 0.001). No patient with a PS of 2–4 had disease progression or intervention. The rates of intervention and mortality did not differ in patients with a PS of 0–1.

### Model and Data Validity

The diagnostic parameters of the model demonstrated adequate internal validity (see Supplementary Table 7 and Supplementary Figures 5 and 6). Assessment of inter- and intra-observer variability across imaging factors showed a good level of agreement (Supplementary Table 8).

## Discussion

In this study of incidental asymptomatic meningiomas, tumor hyperintensity, increasing meningioma volume, proximity to critical neurovascular structures, and peritumoral signal change increased the risk of disease progression within the first 10 years following diagnosis. Based on these factors, patients can be stratified into 3 risk groups, with differing monitoring strategies assigned to each. Patients with an ACCI ≥6 and PS of 2–4 are unlikely to require an intervention for their incidental meningiomas during their estimated lifetimes and thus do not require continued imaging surveillance. These clinical and imaging factors have been grouped to create a prognostic model that can aid clinicians and patients to reach a shared-care decision about management.

### Imaging Factors on MRI and CT

Previous studies have focused on imaging factors that predict meningioma growth and these were also identified in our study. Meningioma hyperintensity is strongly associated with progression^5,20^ along with peritumoral signal change (indicative of vasogenic edema due to breach of the arachnoid plane).^21,22^ The presence of calcification on noncontrast CT was highly correlated with tumor signal intensity on T2/FLAIR and thus was not included as a separate variable in our model. T2, FLAIR, and susceptibility weighted sequencing have all been shown to reliably delineate meningioma-related calcification,^23^ which is a feature of meningiomas that tend to display a much more indolent clinical course.^24,25^ The 2 imaging factors—tumor signal intensity and edema—are not always the main features considered for decision making. Rather, meningioma location and initial volume tend to be key factors for clinicians to recommend early intervention.^19^ While we do not fully agree with this approach, as both surgery and radiotherapy have side effects, we do, however, acknowledge the need to monitor larger meningiomas in certain anatomic locations more closely and this was accounted for in the prognostic model. Loss of “window of curability” is also important to consider. Tumor volume >10 cm^3^ precludes use of stereotactic radiosurgery, and sinus invasion can limit the effectiveness of surgery.^7,8^ Offering treatment before these endpoints are reached makes the assumption that the risk of treatment is lower than the risk of continued surveillance and delayed treatment, which might not be the recommendation of the clinician, but could still be chosen by the patient. Meningiomas in eloquent/skull base locations are also at a higher risk of causing major morbidity compared with convexity meningiomas. Thus, although not statistically significant in multivariate analysis, proximity to critical neurovascular structures was added to the prognostic model. It should, however, be noted that non–skull base meningiomas constitute the majority of those discovered incidentally.^3^ Despite the importance of identifying prognostic factors for growth, there are no studies that examine the duration of follow-up required for incidental meningiomas. Our results indicate that most patients with incidental meningiomas at risk of disease progression requiring consideration of treatment will experience progression-related events within the first 5 years of follow-up.

### Age, Comorbidity, and Performance Status

Patient factors are equally as important as MRI characteristics for clinical decision making. We used the ACCI, which when combined with PS can be used to further stratify the risk of future intervention. Patients were split by ACCI into 2 groups: <6 and ≥6. An ACCI ≥ 6 denotes older patients with comorbidities (eg, an 80-year-old with hypertension and type 2 diabetes mellitus). Although a minority of patients with an ACCI ≥6 experienced disease progression, we did not observe any interventions during prolonged follow-up. The lack of treatment intervention is due to: (i) the high rate of mortality prior to progression (patients were 15 times more likely to die than to receive an intervention at 5 years following diagnosis) and (ii) the threshold for intervention in these patients being much higher. Older patients with comorbidities should not be subject to surgery or radiation solely due to imaging changes, as the risk of morbidity and mortality far outweighs the treatment benefit.^26,27^ For these reasons we propose that patients with an ACCI ≥6 can be discharged from outpatient care with reassurance that their meningiomas are unlikely to cause them problems during their estimated lifetimes. A similar finding was observed in patients with a PS of 2–4, and a similar management strategy could be employed.^28^

### Active Monitoring Strategies

**Fig. 4:**
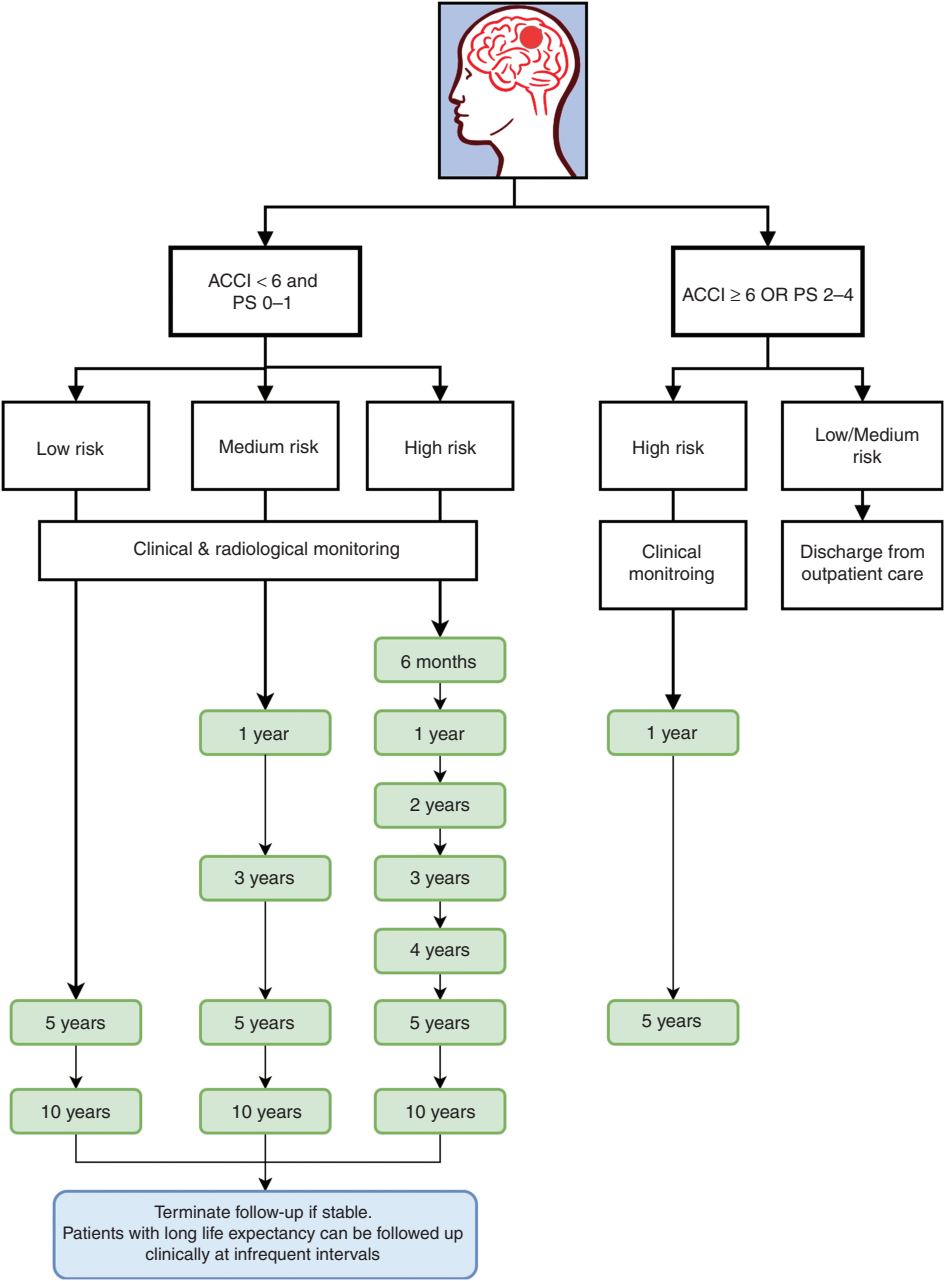
Proposed active monitoring strategies of incidental meningiomas. Time intervals in green boxes are our proposed time points for follow-up.

Comprehensive guidelines for the management of incidental meningioma are lacking,^2^ and there is wide variation in routine clinical practice.^29^ The development of practice parameters should ideally consider individual patient and imaging factors that can aid clinical decision making, similar to those used for unruptured intracranial aneurysms.^30^ Our proposed monitoring strategy is demonstrated in Fig. 4. Based on the prognostic imaging and clinical factors, incidental meningioma patients can be divided into 5 groups. Low- and medium-risk patients with an ACCI ≥6 or PS of 2–4 can be discharged with no subsequent clinical or imaging monitoring but should be counseled about the symptoms that might warrant further examination. Patients in the remaining 4 categories require follow-up but with varying frequencies. High-risk patients with an ACCI ≥6 or PS of 2–4 can be followed clinically with imaging offered on clinical progression only. Low-, medium-, and high-risk patients with an ACCI <6 and a PS of 0–1 can be followed clinically and radiologically but with different time points corresponding to the rates of disease progression (see Fig. 2D). At each appointment, growth rates in concordance with disease progression (AGR ≥ 2 cm^3^/y OR AGR ≥ 1 cm^3^/year+RGR ≥ 30%/y), peritumoral signal intensity, the relationship with neighboring neurovascular structures, and the potential to miss the “window of curability” should be examined. Based on any observed changes, a recommendation for treatment or a decision to continue follow-up can be made and tailored to each patient.

### Beyond 10 Years of Follow-Up

Prognosis beyond 10 years of follow-up for incidental meningioma remains unclear. One study reported growth, defined as >2 mm progression in any unidimensional diameter, beyond 10 years.^31^ However, the results of the joint model used to define disease progression in our study indicated that the rate of tumor growth is of greater clinical importance. Reassessment of ACCI and PS at extended follow-up (beyond 10 y) is also important, since older patients with new comorbidities but who remain radiologically and clinically stable can be safely discharged from outpatient care. Patients with a longer life expectancy, on the other hand, appear to pose an ongoing management dilemma. Based on our observations that imaging changes indicating an intervention are more likely to occur within the first 5 years of follow-up, longer-term imaging surveillance might not be necessary, and instead infrequent clinical monitoring could be adopted.

### Study Limitations

Some limitations of the study should be noted. First, this was a single-center retrospective cohort study with varying nonstandardized follow-up schedules. Nevertheless, appropriate statistical methods were used to account for this. Second, the use of intervention as an endpoint was limited by patient and clinician biases and might have influenced the results of the competing risk analyses. Our tumor board considers the clinical and radiological status of the meningioma, PS, and comorbidities before discussion of the recommended and alternate management strategies with the patient and making a shared-care decision. Due to the retrospective study design, we were unable to ascertain the exact reasons for continued monitoring in cases of progression but surmise that this was due to patient preference (considering personal and social circumstances, employment, loss of driving license for at least 6 months in the UK, risk of posttreatment epilepsy, new neurological deficit, and death). Third, the selection process of a growth endpoint was limited by use of our dataset only and by inevitable competing events such as surgery and radiation, which might have masked the occurrence of the initial composite endpoint. A larger number of events are required to verify our findings and to potentially stratify growth definition by anatomic location. Fourth, we did not have any data on patient quality of life, though it should be noted that most patients remained under follow-up, with the majority reporting no change in clinical symptoms, which supports the notion that most patients with an incidental meningioma lead normal lives—a supposition supported by the limited published quality of life studies.^32,33^ Fifth, patient anxiety and satisfaction with follow-up frequency was not assessed. “Scanxiety” is a well-recognized phenomenon for cancer patients and it is reasonable to assume a similar experience for patients with nonmalignant brain tumors.^34^ The impact on patient well-being of more or less frequent monitoring needs further research. Lastly, socioeconomic status was not assessed. Comorbidity burden and functional status reflect social class and are related to increased risk of mortality.^28,35^ Moreover, access to clinic appointments and treatment is free and available to all patients within the UK’s National Health Service care system and so it was unlikely that social class had an impact on our observation of study endpoints, given the low rate of loss to follow-up (2.7%). However, patients with minimal nonspecific symptoms from lower socioeconomic backgrounds are less likely to present to health care,^36^ which might have reduced the population size and confounded the data.

### Future Work

To keep with reported standards of prognostic models in oncology,^37^ further validation with external retrospective datasets is required. Based on a disease progression risk of 11%, data for a minimum of 1000 patients (100 events^38^) will be needed. Nevertheless, our dataset comprised a large number of patients who are representative of the general meningioma population with associated comorbidity and included a variety of meningioma volumes and locations. Moreover, the parameters associated with internal validation (including discrimination and calibration) demonstrated adequate accuracy. A free online resource has been developed based on our results—the IMPACT calculator (***I***ncidental ***M***eningioma: ***P***rognostic ***A***nalysis Using Patient ***C***omorbidity and MRI ***T***ests) (www.impact-meningioma.com).

## Conclusions

IMPACT offers a personalized active monitoring approach for patients with incidental meningioma and has the potential to reduce the health care costs and patient uncertainty about the need for future treatment. By incorporating clinical and imaging factors into the prognostic model, the need for follow-up and the frequency of imaging can be determined based on the risk of meningioma growth stratified by patient age, comorbidity, and performance status.

## Supplementary Material

noz160_suppl_Supplementary_Material
