# Supplementary material for: A prognostic model to personalize monitoring regimes for patients with incidental asymptomatic meningiomas
Source: Neuro Oncol. 2019 Oct 11;22(2):278–89. doi: 10.1093/neuonc/noz160 (PMC7032634; doi:10.1093/neuonc/noz160)
Supplement: noz160_suppl_Supplementary_Material [file noz160_suppl_supplementary_material.docx]

**Online only supplementary material**

Title: A Prognostic Model to Personalize Monitoring Regimes for Patients with Incidental Asymptomatic Meningiomas

Authors: Islim AI, Kolamunnage-Dona R, Mohan M, Moon RDC, Crofton A, Haylock BJ, Rathi N, Brodbelt AR, Mills SJ & Jenkinson MD

| Supplementary Table S1. Patient demographics and clinical and radiological characteristics | | | | | | |
| --- | --- | --- | --- | --- | --- | --- |
| **Characteristic** |  | **All patients (N=441)** | **Active monitoring (N=385)** | **Discharged (N=50)** | **Surgery (N=6)** | **P** |
| Indication for imaging, N (%) |  |  |  |  |  |  |
| Headache |  | 114 (25.9) |  |  |  |  |
| Cerebrovascular accident |  | 61 (13.8) |  |  |  |  |
| Audiovestibular symptoms |  | 57 (12.9) |  |  |  |  |
| Head trauma |  | 35 (7.9) |  |  |  |  |
| Cognitive deficits |  | 27 (6.1) |  |  |  |  |
| Visual problems |  | 22 (5.0) |  |  |  |  |
| Loss of consciousness |  | 18 (4.1) |  |  |  |  |
| Others |  | 107 (24.3) |  |  |  |  |
| Age (years), mean (SD) |  | 63.3 (12.6) | 62.6 (12.0) | 68.5 (15.9) | 63.8 (10.5) | 0.008*^b^* |
| Sex, N (%) |  |  |  |  |  |  |
| Female |  | 348 (78.9) | 301 (86.5) | 41 (11.8) | 6 (1.7) | 0.365*^c^* |
| Male |  | 93 (21.1) | 84 (90.3) | 9 (9.7) | 0 |  |
| ACCI, N (%) |  |  |  |  |  |  |
| 0-2 |  | 103 (23.4) | 94 (91.3) | 9 (8.7) | 0 | 0.002^c^ |
| 3-5 |  | 212 (48.1) | 193 (91.0) | 15 (7.1) | 4 (1.9) |  |
| ≥6 |  | 126 (28.6) | 98 (77.8) | 26 (20.6) | 2 (1.6) |  |
| WHO PS, N (%) |  |  |  |  |  |  |
| 0-1 |  | 387 (87.8) | 346 (89.4) | 35 (9.0) | 6 (1.6) | 0.001*^c^* |
| 2-4 |  | 54 (12.2) | 39 (72.7) | 15 (27.8) | 0 |  |
| Meningioma count*^a^*, N (%) |  |  |  |  |  |  |
| Single |  | 426 (96.6) | 370 (86.9) | 50 (11.7) | 6 (1.4) | 0.323*^c^* |
| Multiple | 2 | 13 (2.9) | 13 (100) | 0 | 0 |  |
|  | 3 | 1 (0.2) | 1 (100) | 0 | 0 |  |
|  | 4 | 1 (0.2) | 1 (100) | 0 | 0 |  |
| Meningioma volume (cm^3^)*^a^*, median (IQR) |  | 1.6 (0.6-4.0) | 1.7 (0.7-4.2) | 0.7 (0.3-1.4) | 10.6 (4.2-21.6) | <0.001^d^ |
| Meningioma location*^a^*, N (%) |  |  |  |  |  |  |
| Non-skull base | Convexity | 183 (39.9) | 150 (82.0) | 30 (16.4) | 3 (1.6) | 0.478*^c^* |
|  | Parafalcine | 77 (16.8) | 69 (89.6) | 8 (10.4) | 0 |  |
|  | Parasagittal | 36 (8.2) | 35 (97.2) | 0 | 1 (2.8) |  |
|  | Tentorial | 21 (4.6) | 20 (95.2) | 0 | 1 (4.8) |  |
|  | Intraventricular | 5 (1.1) | 5 (100) | 0 | 0 |  |
| Skull base | Sphenoid wing | 45 (9.8) | 39 (86.7) | 5 (11.1) | 1 (2.2) |  |
|  | Posterior fossa-lateral & posterior | 42 (9.2) | 38 (90.5) | 4 (9.52) | 0 |  |
|  | Anterior midline | 34 (7.4) | 32 (94.1) | 2 (5.9) | 0 |  |
|  | Posterior fossa-midline | 16 (3.5) | 15 (93.8) | 1 (6.3) | 0 |  |
| Venous sinus involvement*^a, e^*, N (%) |  |  |  |  |  |  |
| No |  | 291 (63.6) | 246 (84.5) | 42 (14.4) | 3 (1.0) | 0.043*^c^* |
| Yes | Separate (within 10 mm) | 49 (10.5) | 45 (91.8) | 3 (6.1) | 1 (2.0) |  |
|  | In direct contact | 98 (21.4) | 92 (93.9) | 5 (5.1) | 1 (1.0) |  |
|  | Invading | 21 (4.6) | 20 (95.2) | 0 | 1 (4.8) |  |
| Neurovascular structures contact*^a, f^*, N (%) |  |  |  |  |  |  |
| Yes |  | 35 (7.6) | 33 (94.3) | 2 (5.7) | 0 | 0.447*^c^* |
| No |  | 424 (92.4) | 370 (87.3) | 48 (11.3) | 6 (1.4) |  |
| Calcification status*^a^*, N (%) |  |  |  |  |  |  |
| Absent |  | 81 (17.6) | 75 (92.6) | 4 (4.9) | 2 (2.5) | <0.001*^c^* |
| Partial |  | 74 (16.1) | 68 (91.9) | 4 (5.4) | 2 (2.7) |  |
| Diffuse |  | 109 (23.7) | 80 (73.4) | 28 (25.7) | 1 (0.9) |  |
| Tumor signal intensity*^a^*, N (%) |  |  |  |  |  |  |
| Hyper |  | 75 (16.3) | 72 (96.0) | 2 (2.7) | 1 (1.3) | 0.052*^c^* |
| Iso |  | 210 (45.8) | 197 (93.8) | 9 (4.3) | 4 (1.9) |  |
| Hypo |  | 119 (25.9) | 104 (87.4) | 14 (11.8) | 1 (0.8) |  |
| Peritumoral signal intensity*^a^*, N (%) |  |  |  |  |  |  |
| 0-5% |  | 373 (81.3) | 345 (92.5) | 25 (6.7) | 3 (0.9) | <0.001*^c^* |
| 6-33% |  | 16 (3.5) | 16 (100) | 0 | 0 |  |
| 34-66% |  | 13 (2.8) | 11 (84.6) | 0 | 2 (15.4) |  |
| 67-100% |  | 2 (0.4) | 1 (50.0) | 0 | 1 (50.0) |  |
| ACCI=Age-adjusted Charlson comorbidity index; IQR=interquartile range; PS=performance status; SD=standard deviation; WHO=World Health Organization  *^a^*For 459 meningiomas  *^b^*One-way analysis of variance  *^c^*χ2 test  *^d^*Kruskal-Wallis test  *^e^*Venous sinus involvement was noted for 168 meningiomas: superior sagittal sinus (n=95), cavernous sinus (n=35), sigmoid sinus (n=21), transverse sinus (n=15) and the torcula (n=2).  *^f^*Thirty-five meningiomas were in contact with ≥1 critical neurovascular structures and these included: optic apparatus (n=17), internal carotid artery (n=11), basilar artery (n=7), trigeminal nerve (n=4), middle cerebral artery (n=2) and the vertebral artery (n=2). | | | | | | |

| Supplementary Table S2. Primary Kaplan-Meier analyses used to inform the joint longitudinal and survival model of incidental meningioma progression | | | |
| --- | --- | --- | --- |
| **Factor** |  | **HR (95% CI)** | **P** |
| Meningioma hyperintensity |  | 13.5 (6.18-29.4) | <0.001 |
| Calcification |  | 32.2 (4.26-243) | <0.001 |
| Peritumoral signal intensity |  | 6.27 (2.87-13.7) | <0.001 |
| Meningioma size | <1 cm | Reference |  |
|  | 1-2 cm | 2.07 (0.27-16.0) | 0.484 |
|  | 2-3 cm | 6.03 (0.78-46.9) | 0.086 |
|  | ≥3 cm | 16.7 (2.05-136) | 0.009 |
|  | Overall |  | <0.001 |
| Proximity to neurovascular structures |  | 1.99 (0.98-4.03) | 0.050 |
| Location | Non-skull base | Reference |  |
|  | Skull base | 1.78 (0.89-3.57) | 0.103 |
| Number of meningiomas | Single | Reference |  |
|  | Multiple | 1.05 (0.32-3.45) | 0.940 |
| Sex | Female | Reference |  |
|  | Male | 2.39 (1.12-5.08) | 0.020 |
| Age | <50 | Reference |  |
|  | 50-59 | 1.32 (0.48-3.64) | 0.593 |
|  | 60-69 | 0.91 (0.33-2.45) | 0.845 |
|  | 70-79 | 0.71 (0.20-2.53) | 0.598 |
|  | ≥80 | 1.31 (0.15-11.1) | 0.807 |
|  | Overall |  | 0.840 |

| Supplementary Table S3. Joint model parameter estimates | | | |
| --- | --- | --- | --- |
| **Component** | **Parameter** | **Parameter estimate (95% CI)** | **P** |
| Longitudinal | Intercept | 0.14 (-0.04-0.31) | 0.103 |
|  | Time | 0.006 (0.005-0.007) | <0.001 |
|  | Tumor signal intensity | 0.60 (0.23-0.94) | <0.001 |
|  | Peritumoral signal intensity | 1.45 (1.01-1.94) | <0.001 |
|  | Proximity to neurovascular structures | 0.37 (0.11-0.60) | 0.003 |
|  | Location | -0.09 (-0.36-0.14) | 0.483 |
|  | Sex | 0.12 (-0.20-0.39) | 0.469 |
| Survival | Tumor signal intensity | 2.66 (1.81-3.92) | <0.001 |
|  | Peritumoral signal intensity | 1.24 (0.16-2.62) | 0.041 |
|  | Proximity to neurovascular structures | 0.65 (-0.25-1.73) | 0.161 |
|  | Location | 0.66 (-0.29-1.63) | 0.150 |
|  | Sex | 0.23 (-0.88-1.32) | 0.678 |
| Association | Meningioma volume and survival | 0.93 (0.57-1.52) | <0.001 |

| Supplementary Table S4. Kaplan-Meier analyses used to inform the prognostic model | | | |
| --- | --- | --- | --- |
| **Factor** |  | **HR (95% CI)** | **P** |
| Meningioma hyperintensity |  | 13.3 (6.87-25.7) | <0.001 |
| Calcification |  | 22.7 (5.34-96.7) | <0.001 |
| Peritumoral signal intensity |  | 6.67 (3.39-13.1) | <0.001 |
| Meningioma size | <1 cm | Reference |  |
|  | 1-2 cm | 3.01 (0.40-22.6) | 0.284 |
|  | 2-3 cm | 8.53 (1.13-64.7) | 0.038 |
|  | ≥3 cm | 26.7 (3.41-209) | 0.002 |
|  | Overall |  | <0.001 |
| Proximity to neurovascular structures |  | 1.63 (0.90-2.95) | 0.100 |
| Location | Non-skull base | Reference |  |
|  | Skull base | 1.27 (0.69-2.32) | 0.452 |
| Number of meningiomas | Single | Reference |  |
|  | Multiple | 0.97 (0.35-2.74) | 0.962 |
| Sex | Female | Reference |  |
|  | Male | 2.41 (1.29-4.56) | 0.005 |
| Age | <50 | Reference |  |
|  | 50-59 | 1.19 (0.49-2.93) | 0.699 |
|  | 60-69 | 0.87 (0.36-2.07) | 0.747 |
|  | 70-79 | 1.08 (0.40-2.91) | 0.873 |
|  | ≥80 | 1.94 (0.40-9.32) | 0.410 |
|  | Overall |  | 0.820 |

| Supplementary Table S5. Cumulative incidence rates of disease progression and its competing events at diagnosis and at 5 years. | | | | | |
| --- | --- | --- | --- | --- | --- |
| **Event** | **Factor** |  | **At diagnosis** | **5 years** | **P** |
| Disease progression | ACCI | 0-2 | 0.00 | 15.7% | 0.090 |
|  |  | 3-5 | 0.00 | 12.4% |  |
|  |  | >5 | 0.00 | 6.43% |  |
|  | PS | 0-1 | 0.00 | 12.8% | P<0.001 |
|  |  | 2-4 | 0.00 | 0.00 |  |
| HD/LTFU/DDFU | ACCI | 0-2 | 8.49% | 26.0% | <0.001 |
|  |  | 3-5 | 6.81% | 52.8% |  |
|  |  | >5 | 19.5% | 82.0% |  |
|  | PS | 0-1 | 8.66% | 51.0% | P<0.001 |
|  |  | 2-4 | 27.3% | 82.5% |  |
| Intervention | ACCI | 0-2 | 0.00 | 13.5% | 0.009 |
|  |  | 3-5 | 1.81% | 5.76% |  |
|  |  | >5 | 1.50% | 1.50% |  |
|  | PS | 0-1 | 1.48% | 7.27% | P<0.001 |
|  |  | 2-4 | 0.00 | 0.00 |  |
| ACCI=age-adjusted Charlson comorbidity index; DDFU=deceased during follow-up; HD=hospital discharge; LTFU=lost to follow-up; PS=performance status. | | | | | |

| Supplementary Table S6. Cumulative incidence rates of intervention and its competing event at diagnosis and at 5 years. | | | | | |
| --- | --- | --- | --- | --- | --- |
| **Event** | **Factor** |  | **At diagnosis** | **5 years** | **P** |
| Intervention | ACCI | 0-2 | 0.00 | 26.2% | P<0.001 |
|  |  | 3-5 | 1.81% | 9.56% |  |
|  |  | >5 | 1.50% | 2.26% |  |
|  | PS | 0-1 | 1.49% | 13.9% | <0.001 |
|  |  | 2-4 | 0.00 | 0.00 |  |
| Mortality | ACCI | 0-2 | 0.00 | 1.02% | P<0.001 |
|  |  | 3-5 | 0.00 | 9.74% |  |
|  |  | >5 | 0.00 | 33.6% |  |
|  | PS | 0-1 | 0.00 | 10.4% | 0.011 |
|  |  | 2-4 | 0.00 | 45.3% |  |
| ACCI=age-adjusted Charlson comorbidity index; PS=performance status | | | | | |

| Supplementary Table S7. Prognostic model parameters | | | | | |
| --- | --- | --- | --- | --- | --- |
| **Schoenfeld residuals*^a^*** | | **Chambless and Diao’s time depended AUC*^b^*** | | **Concordance statistics*^b^*** | |
| Factor | Test value | Time-point | Value | Type | Value |
| Overall model | 0.964 | 5-years | 0.87 | Harrel’s statistic | 0.89 (95% CI 0.85-0.93) |
| Meningioma volume | 0.662 | 10-years | 0.84 |  |  |
| Tumor signal intensity | 0.824 |  |  |  |  |
| Peritumoral signal intensity | 0.691 |  |  |  |  |
| Proximity to neurovascular structures | 0.637 |  |  |  |  |
| *^a^*Tests were all not statistically significant. The proportional hazards assumption in the prognostic model were therefore not violated**.**  *^b^*Time-dependent AUC values and concordance statistics demonstrated excellent discriminative ability | | | | | |

| Supplementary Table S8. Weighted Kappa values assessing the inter- and intraobserver variability among categorical variables | | |
| --- | --- | --- |
|  | **Weighted Kappa (95% CI)** | |
| **Parameter** | **Inter-observer variability** | **Intra-observer variability** |
| Calcification | 0.82 (0.65-0.99) | 0.85 (0.69-1.01) |
| Tumour signal intensity | 0.80 (0.62-0.98) | 0.83 (0.66-1.01) |
| Peritumoural signal intensity | 0.79 (0.55-1.02) | 1.00 (1.00-1.00) |
| Venous sinus invasion | 0.75 (0.53-0.97) | 0.86 (0.67-1.05) |
|  | **Intraclass correlation coefficient (95% CI)** | |
|  | **Inter-observer variability*^a^*** | **Intra-observer variability*^b^*** |
| Meningioma volume | 0.985 (95% CI 0.966-0.999) | 0.997 (95% CI 0.993-0.999) |
| *^a^*Set to two-way mixed  *^b^*Set to one-way random | | |


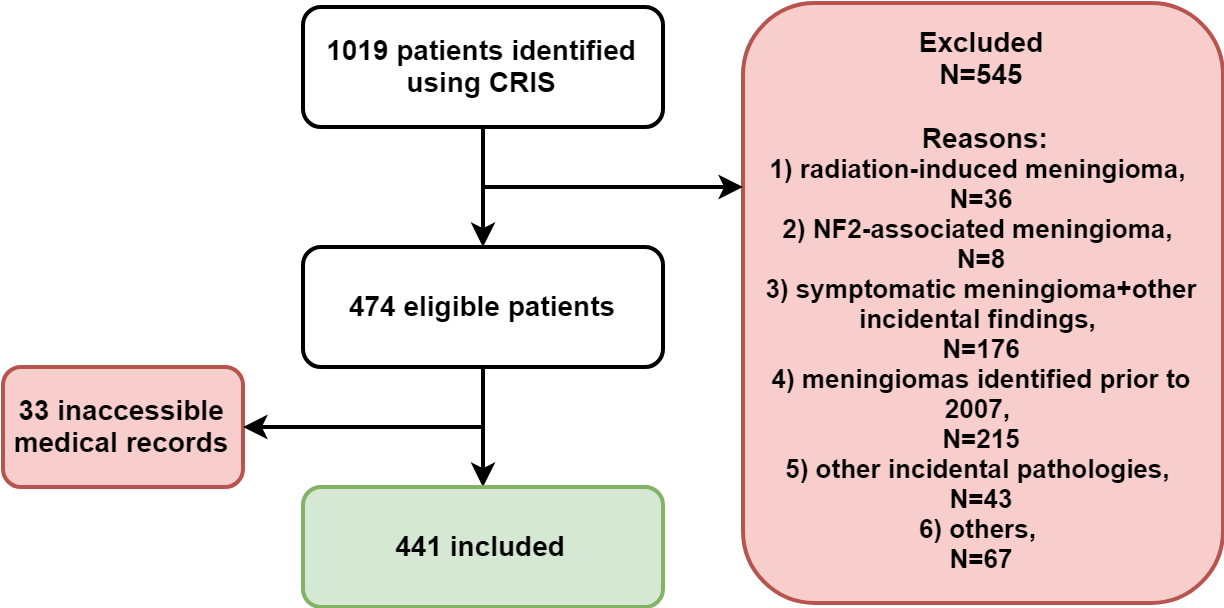


Supplementary Figure S1. Study population selection process


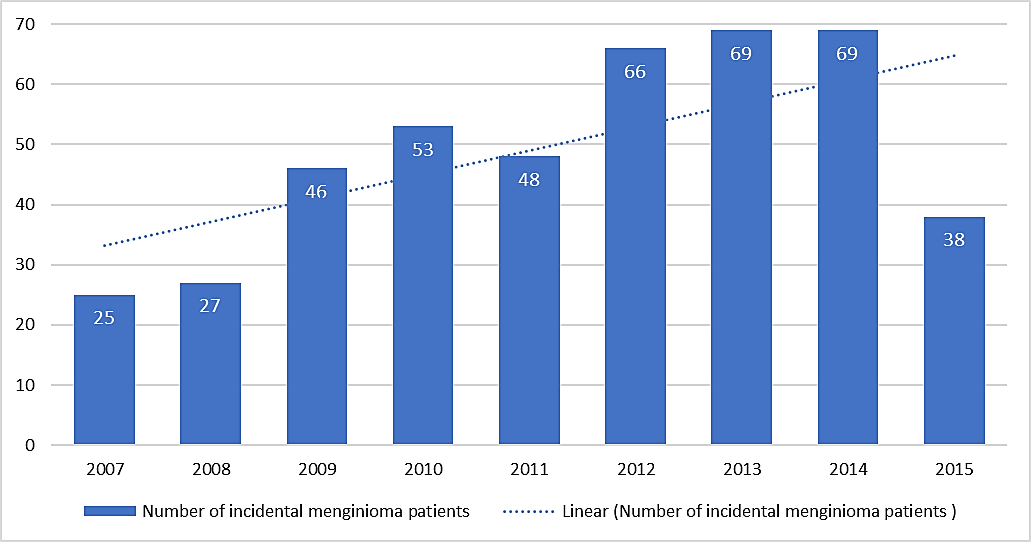


Supplementary Figure S2. Number of incidental meningioma diagnoses per calendar year


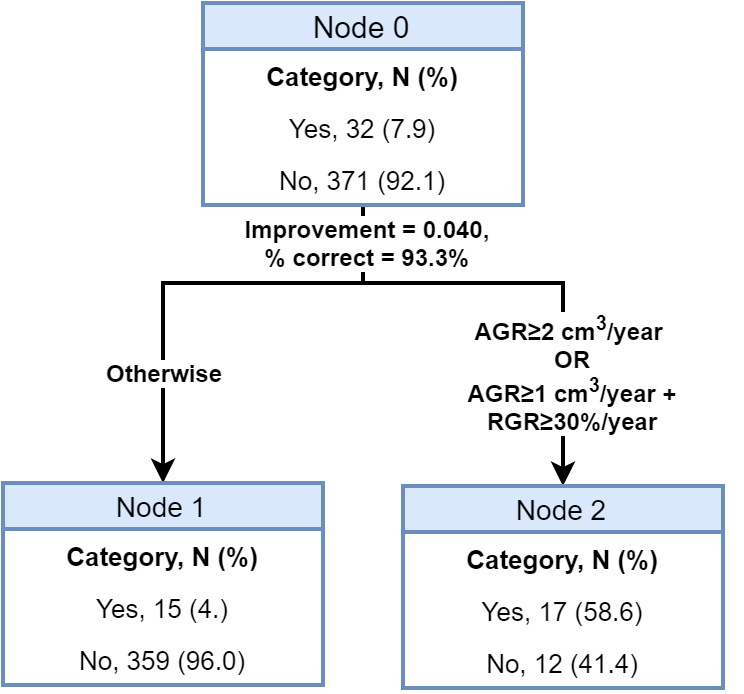


Supplementary Figure 3. CART analysis demonstrating the split in the active monitoring cohort stratified by disease progression and non-progression using AGR≥2 cm^3^/year OR AGR≥1cm^3^/year + RGR≥30%/year to define growth


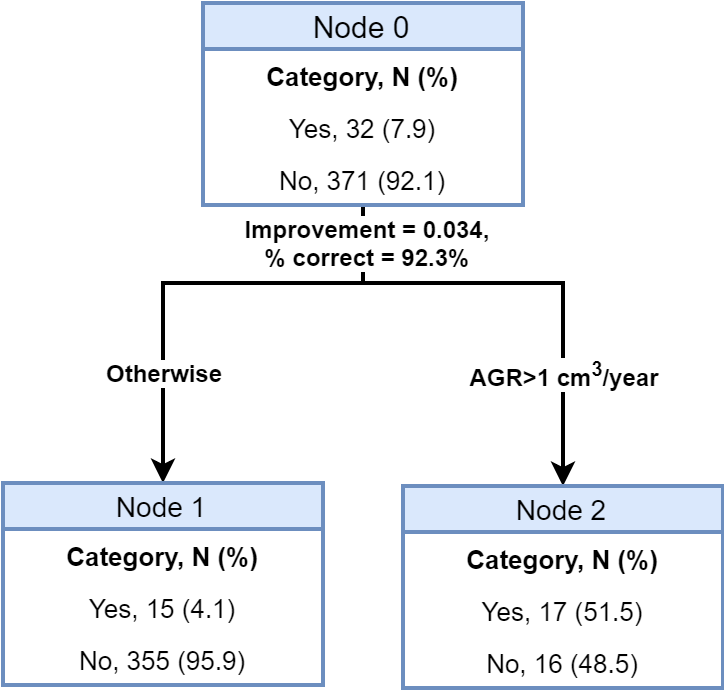


Supplementary Figure 4. CART analysis demonstrating the split in the active monitoring cohort stratified by disease progression and non-progression using AGR>1 cm3/year to define growth


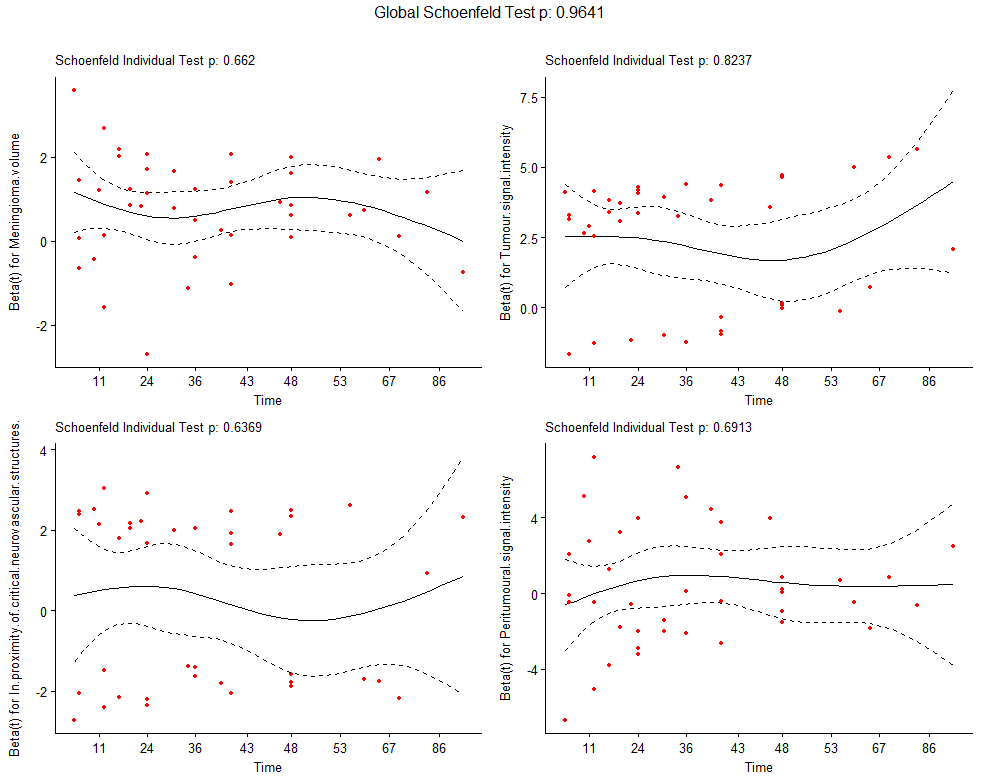


Supplementary Figure S5. Schoenfeld residual plot for each of the covariates. The solid line is a smoothing spline fit to the plot, with the dashed lines representing a ± 2-standard-error band around the fit. None of the plots demonstrated a regular pattern with time, and tests were all not statistically significant. The proportional hazards assumption in model the prognostic model were therefore not violated.

Supplementary Figure S6. (A-B) Calibration plots at 5 and 10 years respectively. Predicted values are plotted on the x-axis and observed values are plotted on the y-axis. The blue bars represent the 95% CIs. Calibration plots demonstrated overall a good level of agreement between the observed and predicted values however some optimism was observed towards the lower probabilities at 5 years and pessimism was noted towards the larger probabilities at 10 years


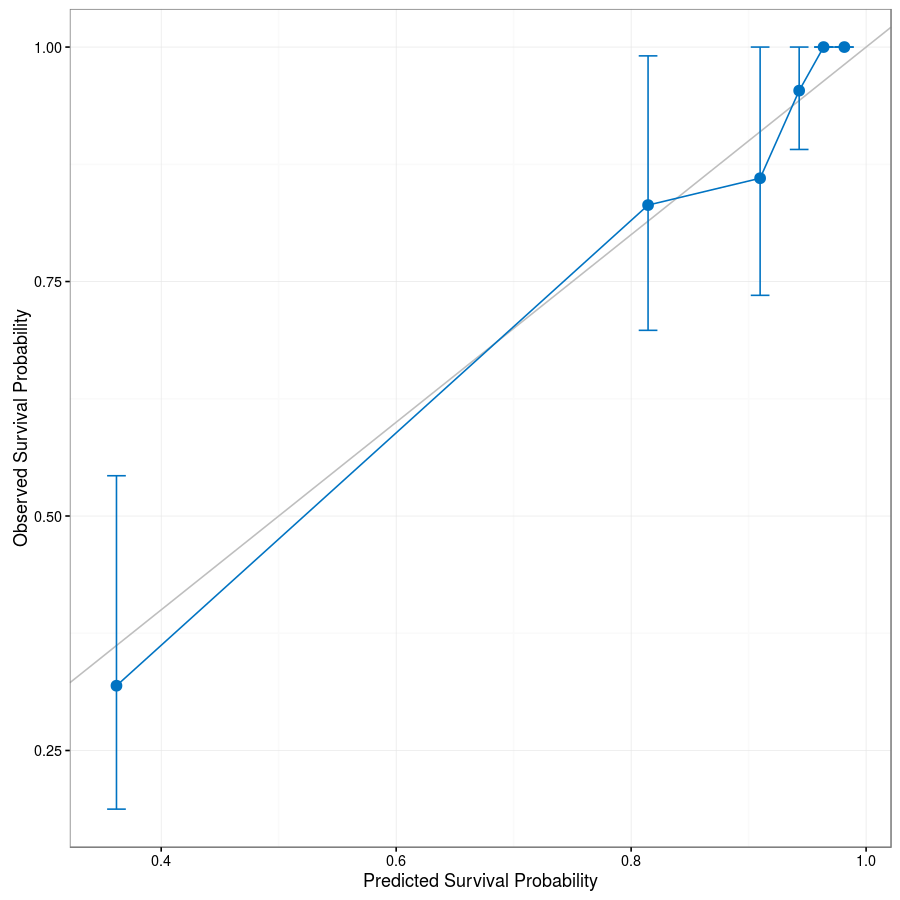

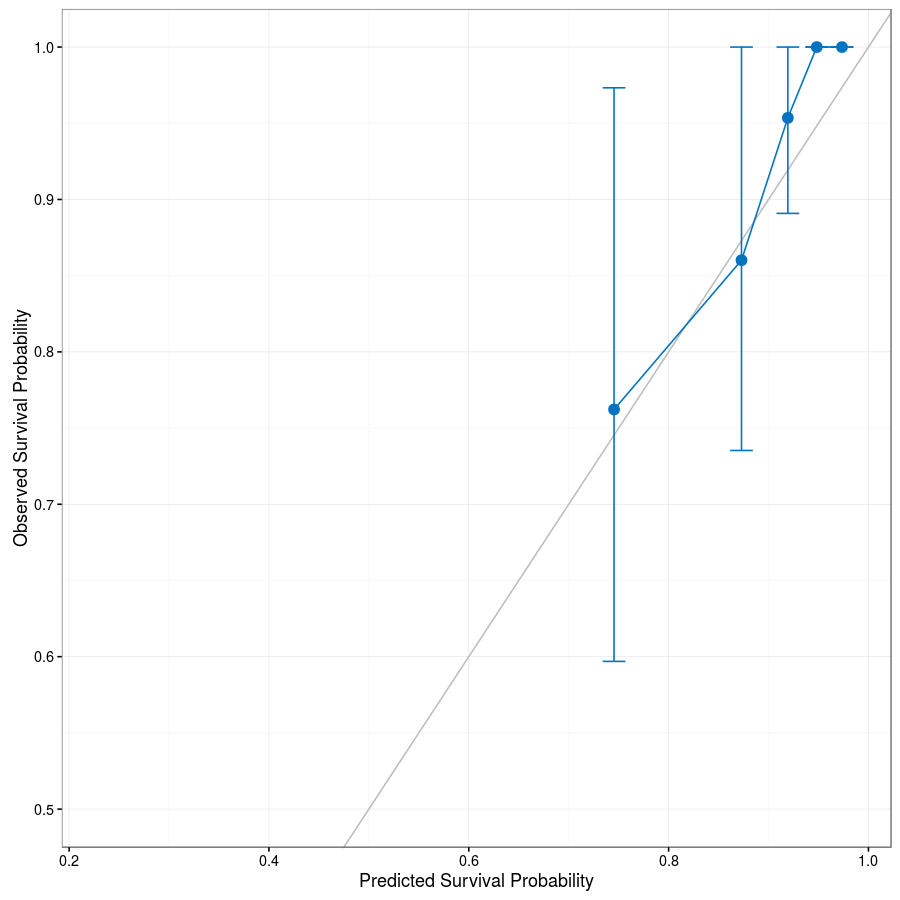


**A**

**B**
